# Prediction of Short-Term Outcome in Acute Superior Vestibular Nerve Failure: Three-Dimensional Video-Head-Impulse Test and Caloric Irrigation

**DOI:** 10.1155/2015/639024

**Published:** 2015-11-16

**Authors:** Holger A. Rambold

**Affiliations:** ^1^Department of Neurology, County Hospitals of Altötting and Burghausen, 84503 Altötting, Germany; ^2^Department of Neurology, University of Regensburg, 93053 Regensburg, Germany

## Abstract

This retrospective study examines acute unilateral vestibular failure (up to seven days after onset) with modern vestibular testing (caloric irrigation and video-head-impulse test, vHIT) in 54 patients in order to test if the short-term outcome of the patients depends on the lesion pattern defined by the two tests. Patients were grouped according to a pathological unilateral caloric weakness without a pathological vHIT: group I; additional a pathological vHIT of the lateral semicircular canal (SCC): group II; and an additional pathological vHIT of the anterior SCC: group III. Patients with involvement of the posterior SCC were less frequent and not included in the analysis. Basic parameters, such as age of the subjects, days after symptom onset, gender, side of the lesion, treatment, and dizziness handicap inventory, were not different in groups I to III. The frequency of pathological clinical findings and pathological quantified measurements increased from groups I to III. The outcome parameter “days spent in the hospital” was significantly higher in group III compared to group I. The analysis shows that differential vestibular testing predicts short-term outcome of the patients and might be in future important to treat and coach patients with vestibular failure.

## 1. Introduction 

Acute unilateral vestibular failure (UVF) is diagnosed by an acute persistent vertigo, spontaneous nystagmus (SPN), and a unilateral vestibular hypofunction of one lateral semicircular canal (SCC) without auditory symptoms [1]. Technically the clinical diagnosis is confirmed by a unilateral pathological head-impulse test (HIT) and/or a unilateral weakness in caloric bithermal irrigation [2]. Modern vestibular techniques using the video-HIT (vHIT) could measure not only the lateral but also, selectively, the vertical (anterior and posterior) SCCs [3, 4]. In addition, the otoliths function could be measured using the cervical and ocular vestibular-evoked potentials (VEMPs) [5]. These modern tests enable us to differentiate lesion patterns, which resemble the innervations pattern of the ampullary nerves and are referred to as inferior, superior, or total vestibular failure [6]. Different lesion pattern classifications have been proposed in recent years based on the results of vHIT and VEMPs [7, 8].

The clinical relevance of the differential vestibular testing with the vHIT is not clear so far. The long-term outcome in various studies is very heterogeneous and it is unclear which parameters might be critical for prognosis and patient management. In recent studies based on a classification of six different lesion patterns, it was shown that the one-year outcome of patients with a lesion pattern of the superior or total vestibular nerve was worse [9]. The short-term outcome of patients with a vestibular neuritis is even more unclear and the time course is scattered [2]. One study showed that an inferior vestibular neuritis had a better prognosis in the time to remission compared to superior or a complete vestibular neuritis form [10].

In previous work we observed that unilateral weakness (UW) of caloric irrigation and vHIT did measure different functions of the VOR [2, 11, 12]. Based on this concept, we retrospectively studied a group of inpatients diagnosed with UVF and analyzed the time of hospitalization and short-term outcome with respect to a classification based on the caloric irrigation and progressively more pathological SCCs identified by the three-dimensional vHIT. We will show that the short-term outcome is worse in more extended lesions of the superior vestibular nerve identified by caloric and vHIT.

## 2. Methods

This is a retrospective study of patients diagnosed with a first-time acute UVF in a time window of up to seven days after symptom onset [1]. Patients exhibiting a hearing loss, signs and symptoms of another peripheral vestibular or brainstem disease (e.g., Menière's disease, horizontal benign paroxysmal positioning vertigo, bilateral vestibular neuritis, and central vestibular disease), or a premedical history of repetitive vertigo were excluded from the study. Patients were included in the analysis if they had a unilateral weakness (UW) over 25% and/or a pathological vHIT. The patients were grouped according* to caloric irrigation and vHIT*: group I, an isolated UW without a pathological vHIT; group II, a unilateral pathological vHIT of the lateral SCC in addition to the pathological UW; group III, a combined pathological vHIT of the lateral and anterior SCCs in addition to the pathological UW; group IV, a pathological vHIT of the lateral and posterior and/or anterior SCC in addition to the pathological UW; and group V, a pathological vHIT of the posterior SCC only. As we found three cases in groups IV and V, they were not included in the analysis.

Patients were routinely examined at the county hospital and underwent a detailed clinical history and standard clinical neurological, oculomotor, and neurovestibular testing performed by the same experienced observer (Holger A. Rambold) [12]. Eye movements were recorded with a commercial binocular video-oculography system (Vestlab 7.1, GN Otometrics, Taastrup, Denmark) to measure the horizontal eye movements; and caloric bithermal testing was performed. UW in the caloric response was quantified according to Jongkees' formula. A value greater than or equal to 25% was pathological according to our normative data. Additionally, the directional preponderance (DP) was measured, which was normal below a value of 30% [12].

The subjective visual vertical (SVV) was measured with the “bucket method” (normative values: ±2°) [13] and the ocular torsion (OT) with a nonmydriatic fundus camera in the two eyes (Topcon TRC NW300; normative values: −2° incyclotorsion to 13.5° excyclotorsion). VEMPs were not elicited or tested in all patients and therefore not analyzed in this paper.

The vHIT (ICS Impulse, GN Otometrics, Taastrup, Denmark, http://www.icsimpulse.com/) was used to test the function of all SCCs. A pathological vHIT was defined as a combination of pathological gain values and correcting saccades for each of the six SCCs [3]. Side difference of the gain was calculated as the ratio of right to left gain difference to right/left gain sum. Additionally the dizziness handicap inventory (DHI) in the German version was used as a subjective parameter [14].

### 2.1. Patients

From August 2012 until December 2014, we selected 59 patients (aged 59 ± 17 years, 34 males, 25 females) diagnosed with UVF (31 right-sided, 28 left-sided), with the first examination up to seven days after symptom onset (3.0 ± 2.2 days). Of these patients four have been published before in [12] and  23 in [11] with respect to the horizontal vHIT and caloric irrigation. In addition to the clinical examination, the testing always included a three-dimensional vHIT and a caloric bithermal irrigation, a VOG-testing (SPN in the dark in sitting position), the SVV, and a fundus photography on the same day. All patients were treated after the vestibular testing with dimenhydrinate to relieve autonomous symptoms, with oral corticoids, prednisolone, one mg/kg of body weight if necessary [15], and with physiotherapy according to a standardized protocol. Clinical details and basic measurements at first examination are shown in Table 1.

Patients were dismissed from the hospital if they were able to ambulate independently without falling during clinical gait test with eyes open on a firm floor. At the same time, they were routinely asked if they still had symptoms, such as dizziness, vertigo, unsteadiness, or oscillopsia with fast head movements or if they were symptom-free.

### 2.2. Statistical Design

As our data could not be described by a normal distribution we used the Kruskal-Wallis test for the group comparison of quantitative data (Table 2) and Fisher's exact test for the group comparison of qualitative data (Table 1). Linear correlation analysis of the form out(*t*) = intercept + slope*∗t* was performed and the correlation coefficient of determination (*r* ^2^) used. Significant difference of the slope from zero was tested by the *t*-test (Matlab, The Mathworks Inc., USA).

## 3. Results

### 3.1. Basic Parameters

Groups I to III were not different in the basic parameters such as age of subjects, days of admission after symptom onset (Table 2), gender, side of the lesion, and prednisolone and dimenhydrinate treatment (Tables 1(a) and 1(c)). The initial DHI was not significantly different between the three groups, but there was an increasing trend from groups I to III (Table 2). The frequency of* clinically* observed spontaneous and head shaking nystagmus (nystagmus after 20 s of horizontal head shaking with eye closed) and of pathological Romberg test increased from groups I to III (Table 1). Similarly, but with less significance, the frequency of the pathological Fukuda-Stepping test increased from groups I to III (s. Table 1(b)). A clinically observed gait disturbance was very similar in groups I to III.

### 3.2. Vestibular Tests

Vestibular tests coded as pathological were expressed as frequency per group and compared (Table 1(d)). The frequency of pathological SVV and OT increased from groups I to III. Quantified values of the vestibular tests are presented for the three groups in Table 2. There was an increase in the value of the SVV, OT, and slow-phase velocity of SPN from groups I to III, with a statistical significance between groups I and III. The value of UW was increased in group III compared to groups I and II. The DP was significantly increased from groups I to III, comparable to the increase in SPN.

The vHIT gain values of the contralesional SCC were within normal limits. Ipsilesional vHIT gains of the lateral SCC were reduced in groups II and III and gains of the anterior SCC in group III, as expected. There were no differences in stimulus velocities of the vHIT to the contra- and ipsilesional side and in the three groups for the lateral and posterior SCC. The vHIT of the anterior SCC had slightly higher stimulus velocity in group II for the ipsilesional side. Nevertheless, the obtained velocity values are within realistic clinical limits (head peak velocities during lateral SCC stimulation was 200–230°/s and during vertical SCC stimulation 123–158°/s; Table 2). Frequency of overt and covert saccades for the lateral vHIT was not different in group II versus III.

### 3.3. Outcome

The short-term outcome parameter, days spent in the hospital, was significantly higher in group III (6.0 ± 1.5 days) compared to group I (3.6 ± 1.5 days). Group II (5.1 ± 3.0 days) was not different from groups I and III (Table 2). Most patients had symptoms at dismissal (75%). Frequencies of symptoms at dismissal were lower in groups I (71%) and II (72%) compared to group III (83%), but without any statistical significance (Table 1(e)). We did not find any significant linear correlation between the outcome parameter “days spent in the hospital” and the quantified parameters of vestibular function as, for example, SPN, OT, SVV, UW, and DP, indicating that those parameters do not predict the outcome in our data.

## 4. Discussion

Patients with different lesion patterns based on caloric irrigation and three-dimensional vHIT predict a different short-term outcome defined by the time of hospitalization. Patients without symptoms at dismissal were statistically not different between the groups but showed a trend towards less symptoms in groups I and II compared to group III.

We are aware that our results on the short-term outcome could be influenced by different parameters, for example, the social and cultural background of the patients and the general nursing and management of the ward in our hospital, which could not be controlled. Nevertheless, in our consistent setting with standardized treatment algorithms we could show an effect of the extent of the lesion on the parameter, days the patients spend in hospital, in this retrospective analysis. This might be economically important to plan and manage patients on the ward.

### 4.1. Limitations

There are some limitations, as in every retrospective study. This study includes routine patient data and might have a selection bias due to the health system organization and the patients sent to our specialized vertigo clinic. In this study, we were careful that the vHIT glasses did not slip on the patient's head and that traces with artifacts as blinks were excluded. The vHIT is sensitive to the examiner. In particular the maximal stimulus velocity applied is critical. Our data shows that we reached high enough stimulation velocities around 200–230°/s horizontally and 125–158°/s vertically to be within the recommended range [3]. Indeed, higher stimulus velocities might show more pathological values, but often such velocities could not be reached in the routine examination.

### 4.2. Comparison to Previous Studies

In contrast to previous studies, we focused on the short-term outcome and on a group of patients with a superior vestibular nerve lesion pattern. Vestibular loss consistent with a lesion pattern of the inferior and total vestibular nerve was not included in the study due to insufficient patient numbers. In another study a very similar analysis was performed in Japan which compared the superior, inferior, and total vestibular lesion pattern in vestibular neuritis (VN) [10], showing that the latter had a longer time to remission. They showed a trend for the time of hospitalization increasing from inferior to superior to total VN, which was not significant. For the superior nerve pattern, we had slightly shorter but comparable data of hospitalization. This is in line with worse short-term and long-term outcome with increasing lesion load [8, 10].

### 4.3. Lesion Load

The frequency of pathological clinical signs and the increase in pathological vestibular assessments was increased from groups I to III indicating an increase in the extent of the vestibular lesion pattern as reported before [11, 12]. The gain of the vHIT of the lateral SCC decreased from groups I to III, with pathological gain values in groups II and III. Similarly we found signs of static vestibular imbalance measured as SVV and OT and of dynamic imbalance measured as SPN, HSN, or DP which increased from groups I to III, very similar to a previous publication [11, 12]. This supports an increasing lesion load from groups I to III.

If the different lesion patterns (groups I to III) resemble different etiologies or disease remains unclear. The etiology of acute vestibular failure is controversial and might be caused by a viral or vascular genesis [1]. By clinical means, we excluded all other peripheral, central, and repetitive vestibular diseases. Group I was diagnosed based on a unilateral weakness in caloric testing in the acute stage. This group is not of central origin by clinical means and actual diagnostic standards.

The difference of caloric irrigation and vHIT has been discussed in previous papers before [2, 12] and is still under debate. It is known from previous studies that pathological lateral (v)HITs were observed with a UW higher than 43% [12, 16]. This is consistent with presented data here with an average of 41% UW in group I (caloric only) and higher values in groups II (48%) and III (63%). In recent studies on Menière's disease (MD) it was hypothesized that the dissociation of a pathological caloric and normal vHIT might be a consequence of the physical enlargement of the membranous duct in the hydropic labyrinths [17]. According to clinical criteria we excluded patients with MD, but an isolated endolymphatic hydrops in the labyrinth could be possible.

## 5. Conclusion

The extent of the lesion measured by caloric irrigation and vHIT has a direct impact on time of hospitalization. Caloric only lesion and additional lesion of the lateral SCC have a better short-term outcome than similar lesions with anterior canal involvement.

## Figures and Tables

**Table:** (a) Frequency of basic parameters

	%	%	%	*p* Fisher's exact		
	Group I	Group II	Group III	I versus II	I versus III	II versus III
Males	50	61	67	0.721	0.492	0.754
Comorbidity	50	50	13	1.000	**0.023**	**0.016**
Lesion right side	35	61	54	0.285	0.328	0.757

**Table:** (b) Frequency of pathological clinical signs

	%	%	%	*p* Fisher's exact		
	Group I	Group II	Group III	I versus II	I versus III	II versus III
HSN	43	87	100	**0.021**	**0.000**	0.167
SPN	21	65	87	**0.029**	**0.000**	0.134
Romberg test	14	33	71	0.390	**0.002**	**0.041**
Fukuda-Stepping test	54	87	95	0.096	**0.010**	0.571
Gait disturbance	64	88	91	0.198	0.084	1.000

**Table:** (c) Frequency of treatment

	%	%	%	*p* Fisher's exact		
	Group I	Group II	Group III	I versus II	I versus III	II versus III
Prednisolone	15	22	29	1.000	0.446	0.731
Dimenhydrinate	31	38	30	0.507	0.658	0.79

**Table:** (d) Frequency of pathological tests

	%	%	%	*p* Fisher's exact		
	Group I	Group II	Group III	I versus II	I versus III	II versus III
UW %	100	100	100	1.000	1.000	1.000
vHIT LC %	0	100	100	**0.000**	**0.000**	1.000
vHIT AC %	0	0	100	1.000	**0.000**	**0.000**
vHIT PC %	0	0	0	1.000	1.000	1.000
SVV %	31	75	79	**0.027**	**0.006**	1.000
OT %	10	50	77	0.087	**0.001**	0.098

**Table:** (e) Frequency of symptoms at discharge

	%	%	%	*p* Fisher's exact		
	Group I	Group II	Group III	I versus II	I versus III	II versus III
	71	72	83	1.000	0.433	0.462

Frequency per group is given in percentage and *p* values of Fisher's exact probability test obtained by intergroup comparison. Significant different *p* values are indicated in bold numbers.

HSN: head shaking nystagmus, SPN: spontaneous nystagmus in the dark, UW: unilateral weakness of caloric irrigation, DP: directional preponderance of caloric irrigation, vHIT: video-head-impulse test, LC: lateral SCC, AC: anterior SCC, PC: posterior SCC, SVV: subjective visual vertical, OT: ocular torsion.

**Table 2:** Quantitative data.

Variable		Group I	Group II	Group III	Significant differences		
		mean ± std	mean ± std	mean ± std	I versus II	I versus III	II versus III
Basic							
Age [years]		64 ± 17	63 ± 17	53 ± 16			
Days to admission		2.6 ± 2.0	3.2 ± 2.6	2.7 ± 1.9			
Days in clinic		3.6 ± 1.5	5.1 ± 3.0	6.0 ± 1.5		×	
DHI		32 ± 20	39 ± 29	50 ± 27			
Tests							
SVV [°]		0.3 ± 1.65	3.3 ± 5.2	7.1 ± 5.3		×	
OT [°]	ipsi	7.5 ± 2.9	13.2 ± 7.1	15.8 ± 7.6		×	
OT [°]	contra	6.6 ± 4.3	1.1 ± 8.8	−3.5 ± 6.2		×	
SPN [°/s]		−0.6 ± 3.5	−3.3 ± 4.2	−6.4 ± 5.7		×	
Caloric							
UW [%]		41 ± 20	48 ± 16	63 ± 25		×	
DP [%]		34 ± 39	83 ± 61	152 ± 82		×	
vHIT							
Gain LC	ipsi	0.97 ± 0.16	0.65 ± 0.29	0.36 ± 0.18		×	×
Gain LC	contra	0.96 ± 0,12	1.03 ± 0.23	0.87 ± 0.17			
Gain AC	ipsi	0.93 ± 0.15	1.02 ± 0.21	0.53 ± 0.18		×	×
Gain AC	contra	1.03 ± 0.20	0.99 ± 0.25	1.02 ± 0.20			
Gain PC	ipsi	1.05 ± 0.28	1.05 ± 0.28	1.03 ± 0.19			
Gain PC	contra	1.05 ± 0.19	1.04 ± 0.29	0.97 ± 0.25			
SD LC		0.07 ± 0.05	0.27 ± 0.05	0.47 ± 0.16		×	×
SD AC		0.08 ± 0.04	0.11 ± 0.05	0.34 ± 0.15		×	×
SD PC		0.13 ± 0.10	0.11 ± 0.09	0.16 ± 0.10			
LC Vel [°/s]	ipsi	218 ± 37	216 ± 39	232 ± 35			
LC Vel [°/s]	contra	205 ± 39	224 ± 40	218 ± 39			
AC Vel [°/s]	ipsi	129 ± 27	123 ± 27	158 ± 31			×
AC Vel [°/s]	contra	133 ± 25	137 ± 26	137 ± 29			
PC Vel [°/s]	ipsi	125 ± 26	128 ± 29	138 ± 26			
PC Vel [°/s]	contra	127 ± 26	131 ± 28	138 ± 31			

Quantified data are shown for groups I to III as mean standard deviation (std). To the right the significant differences (*p* < 0.05, Kruskal-Wallis test) of intergroup comparison are indicated by an ×.

DHI: dizziness handicap inventory, SVV: subjective visual vertical, OT: ocular torsion, SPN: slow phase velocity of the SPN in the dark, UW: unilateral weakness of caloric irrigation, DP: directional preponderance of caloric irrigation, vHIT: video-head-impulse test; LC: lateral SCC, AC: anterior SCC, PC: posterior SCC, Vel: maximal stimulus velocity applied during the vHIT, SD: side difference of the vHIT. The measurement side is given in respect to the lesion side (ipsi: ipsilesional, contra: contralesional).
